# First Draft Genome Sequence of a *Mycobacterium gordonae* Clinical Isolate

**DOI:** 10.1128/genomeA.00638-16

**Published:** 2016-06-30

**Authors:** V. Ustinova, T. Smirnova, K. Blagodatskikh, D. Varlamov, D. Sochivko, E. Larionova, S. Andreevskaya, I. Andrievskaya, L. Chernousova

**Affiliations:** a Microbiology Department, Central Tuberculosis Research Institute RAMS, Moscow, Russia; b All-Russia Research Institute of Agricultural Biotechnology, Moscow, Russia; c R&D company Syntol, Moscow, Russia

## Abstract

Here, we report the first draft genome sequence of the clinically relevant species *Mycobacterium gordonae*. The clinical isolate *Mycobacterium gordonae* 14-8773 was obtained from the sputum of a patient with mycobacteriosis.

## GENOME ANNOUNCEMENT

*Mycobacterium gordonae* is a nontuberculous mycobacterium, named for Ruth E. Gordon, who first isolated the species. *M. gordonae* grows slowly, forming smooth, yellow- or orange-colored colonies. Of all the mycobacteria species, *M. gordonae* is one of the least pathogenic. Its isolation from samples of sputum obtained from patients is typically regarded as a contamination. Such a contamination can be associated with a patient’s use of tap water for mouth rinsing or for drinking to obtain better expectoration just before preparing a test sample (1). Despite its nonvirulent nature, there have been reports of clinically significant diseases caused by *M. gordonae*, including disseminated infections (2), urogenital tract diseases (3), gastrointestinal tract infections (4), soft tissue damage (5), and respiratory and pulmonary infections (6–8).

We report here the first draft genome sequence of *M. gordonae* clinical isolate 14-8773, which was obtained from the sputum of a patient admitted to a tuberculosis hospital with suspected pulmonary tuberculosis (confirmed by lung abnormalities in a chest X ray and positive test results from an acid-fast stain). Two isolates were obtained on Löwenstein-Jensen medium from two different portions of sputum. The genomic DNA of *M. gordonae* was extracted by the guanidiniumthiocyanate DNA isolation method with sorbtion on magnetic beads and purified by phenol-chloroform-isoamyl alcohol separation followed by ethanol precipitation. Species identification of the two cultures was carried out using the GenoType CM test system (Hain Lifescience, Germany). The identification result for clinical isolate 14-8773 was confirmed by 16S rRNA gene sequencing.

Paired-end libraries with an average insert size of 500 bp were generated using the Illumina Nextera XT DNA sample preparation kit according to the manufacturer’s protocol. Sequencing was performed on the Illumina MiSeq platform with a 2 × 250 paired-end run (theoretical coverage of 200×). The obtained reads were analyzed and quality-checked using FastQC version 0.11.3 (9), and subsequently trimmed. Illumina adapters, bases with quality lower than Q30, N bases, and reads shorter than 50 bp were removed using Trimmomatic version 0.33 (10). Reads were assembled using SPAdes version 3.5.0 (11) with the “--careful” option and with *k* values of 21, 33, 55, 77, 99, and 127. The final assembly consisted of 377 contigs comprising 7,552,315 bp with an *N*_50_ of 61,403 bp and a GC content of 66.76%. Annotation was carried out using the NCBI Prokaryotic Genome Annotation Pipeline (PGAP) (12). PGAP annotation resulted in 6,889 predicted genes, 6,413 coding sequences, 424 pseudogenes, one 16S rRNA, one 23S rRNA, one 5S rRNA, 48 tRNAs, and one noncoding RNA.

This draft genome sequence and its functional annotation analysis will help to unravel the phylogeny of *Mycobacterium gordonae*, as well as its metabolic and pathogenic potential.

### Nucleotide sequence accession numbers.

This whole-genome shotgun project has been deposited at DDBJ/EMBL/GenBank under the accession number LKTM00000000. The version described in this paper is the first version, LKTM01000000.
